# Anti-Overturning Fully Symmetrical Triboelectric Nanogenerator Based on an Elliptic Cylindrical Structure for All-Weather Blue Energy Harvesting

**DOI:** 10.1007/s40820-022-00866-w

**Published:** 2022-05-11

**Authors:** Dujuan Tan, Qixuan Zeng, Xue Wang, Songlei Yuan, Yanlin Luo, Xiaofang Zhang, Liming Tan, Chenguo Hu, Guanlin Liu

**Affiliations:** 1 Department of Applied Physics, State Key Laboratory of Power Transmission Equipment & System Security and New Technology, Chongqing University, Chongqing, 400044 People’s Republic of China; 2 Center On Nanoenergy Research, School of Physical Science and Technology, Guangxi University, Nanning, Guangxi 530004 People’s Republic of China

**Keywords:** Anti-overturning, Fully symmetrical, Elliptic cylindrical, Triboelectric nanogenerator, Wave energy

## Abstract

Triboelectric nanogenerators (TENGs) have shown promising potential for large-scale blue energy harvesting. However, the lack of reasonable designs has largely hindered TENG from harvesting energy from both rough and tranquil seas. Herein, a fully symmetrical triboelectric nanogenerator based on an elliptical cylindrical structure (EC-TENG) is proposed for all-weather blue energy harvesting. The novel elliptical cylindrical shell provides a unique self-stability, high sensitivity to wave triggering, and most importantly, an anti-overturning capability for the EC-TENG. Moreover, benefiting from its internal symmetrical design, the EC-TENG can produce energy normally, even if it was overturned under a rude oscillation in the rough seas, which distinguishes this work from previous reported TENGs. The working mechanism and output performance are systematically studied. The as-fabricated EC-TENG is capable of lighting 400 light-emitting diodes and driving small electronics. More than that, an automatic monitoring system powered by the EC-TENG can also monitor the water level in real-time and provide an alarm if necessary. This work presents an innovative and reliable approach toward all-weather wave energy harvesting in actual marine environments.

**Supplementary Information:**

The online version contains supplementary material available at 10.1007/s40820-022-00866-w.

## Highlights

- The novel elliptical cylindrical structure (EC) provided an excellent self-stability, high sensitivity in small agitations, and most importantly, a distinctive anti-overturning capability for the EC-triboelectric nanogenerator (EC-TENG).
- Benefitting from the fully symmetrical design, the EC-TENG could maintain the original output after being overturned under extreme conditions, distinguishing itself from previous TENGs.
- Two built-in TENGs designed for use in rough seas and tranquil seas improved the collection efficiency for all-weather wave energy.

**Supplementary Information:**

The online version contains supplementary material available at 10.1007/s40820-022-00866-w.

## Graphical abstract

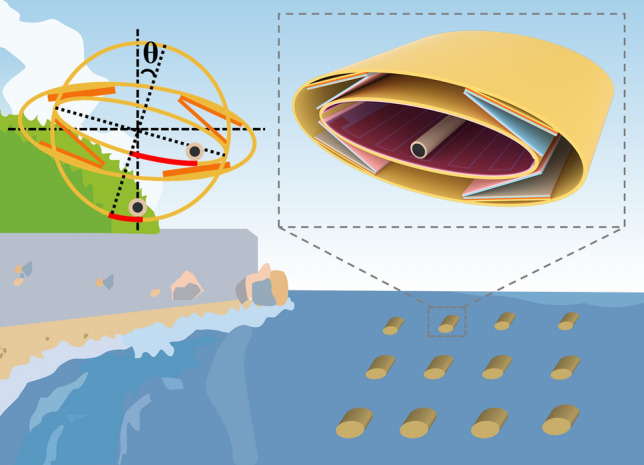

## Introduction

The ocean covers more than 70% of the earth's surface, offering vast amounts of renewable energy [1]. Ocean wave energy is regarded as one of the most convenient renewable energies to be utilized directly due to the characteristics of being inexhaustible and widely distributed [2, 3]. Owing to its high energy density and clean feature [4, 5], ocean wave energy has raised the potential to compete with the currently used fossil energy. To date, most wave energy harvesters rely on electromagnetic generators (EMGs), which are heavy, costly, and easy to be corroded in water [6, 7]. Moreover, EMGs also suffer insufficient energy conversion efficiencies at low wave frequency and randomly moving direction [8]. Hence, a lightweight, corrosion-resistant, cost-effective, highly efficient energy converter for wave energy harvesting is highly desirable, and triboelectric nanogenerators (TENGs) could be a solution to this problem [9–11].

TENG, whose theoretical origin is Maxwell’s displacement current [12], has been proven to be a powerful technology for converting low-frequency mechanical energy into electricity based on the coupling of triboelectrification and electrostatic induction [13–19]. Since its birth, various TENGs have been developed for wave energy harvesting, such as tower-like TENG [20], pendulum-inspired TENG [21], spherical-shell structures [22–24], wavy structures [25–27], spring-assisted structures [28–30], bionic structures [31–33], hybrid generators [34–38], and other types of combined structure designs [39–41]. Most previously reported TENGs can achieve optimal energy output in the rough seas, but they perform poorly in the halcyon seas, which is common in the actual environment. Moreover, under extreme wave conditions, most of the previous TENGs would lose their optimal working states due to excessive rotation or overturning, thus resulting in a sharp decrease in output. Although this drawback can be obviated by adding additional tumbler-type accessories [42] or limiting cables [43], the device would be more bulky and costly, and the response of the device to waves would also be weakened. Therefore, a novel design of TENG with ideal performance in both tranquil and choppy seas is urgently needed to further boost TENGs for the blue energy dream.

In this work, a fully symmetrical TENG with an elliptic cylindrical swing structure (named EC-TENG) is designed to effectively overcome the above-mentioned problems of previous TENG-based wave energy harvesters. The EC-TENG is composed of two coaxial elliptical cylindrical shells, where the inner TENG adopts a freestanding mode using a steel bar as the rolling element, while the outer one has four identical contact-separation mode TENGs. The two elliptical cylindrical shells are connected by a rod bearing to allow the inner shell to swing in the outer one if the EC-TENG leaned. Benefitting from the elliptical cylindrical design, the steel rod of the inner TENG demonstrates a nimble rolling activity under a tiny excitation, efficiently capturing the water-wave energy in tranquil seas. In addition, the elliptic cylindrical shell has a self-stabilization ability that guarantees an anti-overturning capability even under rough external triggering. Even if the EC-TENG is overturned by extremely powerful waves, it will still work normally with no reduction in its output owing to the completely symmetrical structure. The working mechanism and output performance have been systematically studied. Experimental result demonstrates the EC-TENG can light up 400 LEDs and power some small electronics. Besides, a real-time water level monitoring and alarm system are also achieved by the EC-TENG. The EC-TENG can be easily fabricated, maintaining a high sensitivity to wave triggering, and can provide an effective approach for collecting all-weather blue energy.

## Experimental Section

### Fabrication of EC-TENG

Elliptic cylindrical shells made of polylactic acid (PLA) were prepared using a 3D printer based on the parameters shown in Fig. S7. The lengths of the inner shell and the outer shell were 102 and 104 mm respectively. For the inner TENG, a steel bar (7 mm in diameter and 95 mm in length) was inserted into a PTFE tube (internal diameter: 7 mm; external diameter: 9 mm; length: 95 mm) as the rolling element. Conductive ink was used to fabricate interdigital electrodes on a polyethylene terephthalate (PET) substrate (thickness: 0.1 mm) using the silk screen printing technic, and two copper wires were connected to these two electrodes on the edge as external electrodes. Then a nylon film (25 μm in thickness, purchased from a local market) with back glue was attached to the printed interdigital electrodes. Finally, two identical PET/interdigital electrodes/nylon films were symmetrically attached to the upper and lower inner sides of the inner elliptical cylindrical shell. For the outer TENGs, four pairs of acrylic sheets (40 × 100 × 1 mm^3^) and PET films (40 × 100 × 0.5 mm^3^) were prepared using a laser cutting machine. An aluminum (Al) film (30 μm in thickness) was then attached to one side of the acrylic as an electrode, and then a PTFE film (50 μm in thickness, purchased from Sigma-Aldrich) was adhered to the Al film as a tribo-layer. To further improve the triboelectric charge density, the PTFE film was treated under inductive coupled plasma (ICP, SENTECH SI500) for 2 min to obtain a nanostructured surface. Next, an Al film (30 μm in thickness) was fastened to the PET film (0.5 mm thickness) as another electrode and tribo-layer. The surface characters of triboelectric materials used in this work were characterized by a field emission scanning electron microscopy (FESEM, JEOL 7500, Japan), and the results are shown in Fig. S8. Kapton tape was used to mount the PTFE/Al/acrylic and Al/PET with the tribo-layers in the inner sides and allowed the TENG to open in a V-shape. After that, four V-shaped TENGs were fastened symmetrically between the inner and outer shells. Finally, the open ends of the outer and inner shells were sealed by elliptical acrylic plates, and a thin steel rod was passed through their axes, allowing the inner shell to swing freely in the outer one and be at the center of the device in the equilibrium position. When the inner shell swung, the outer TENGs would separate and contact periodically.

### Device Characterization

A Keithley 6514 system electrometer based on the LabVIEW software platform was used to measure the output performance of the EC-TENG. A finite-element simulation using COMSOL Multiphysics software was used to calculate the potential distribution of the TENG. A stepping motor (86HB250-118B) was used to agitate the device for experiments in the atmosphere. Finally, a wave-making pump was used to generate water waves for the experiments in water.

## Results and Discussion

### Design and Working Principle of EC-TENG

**Fig. 1:**
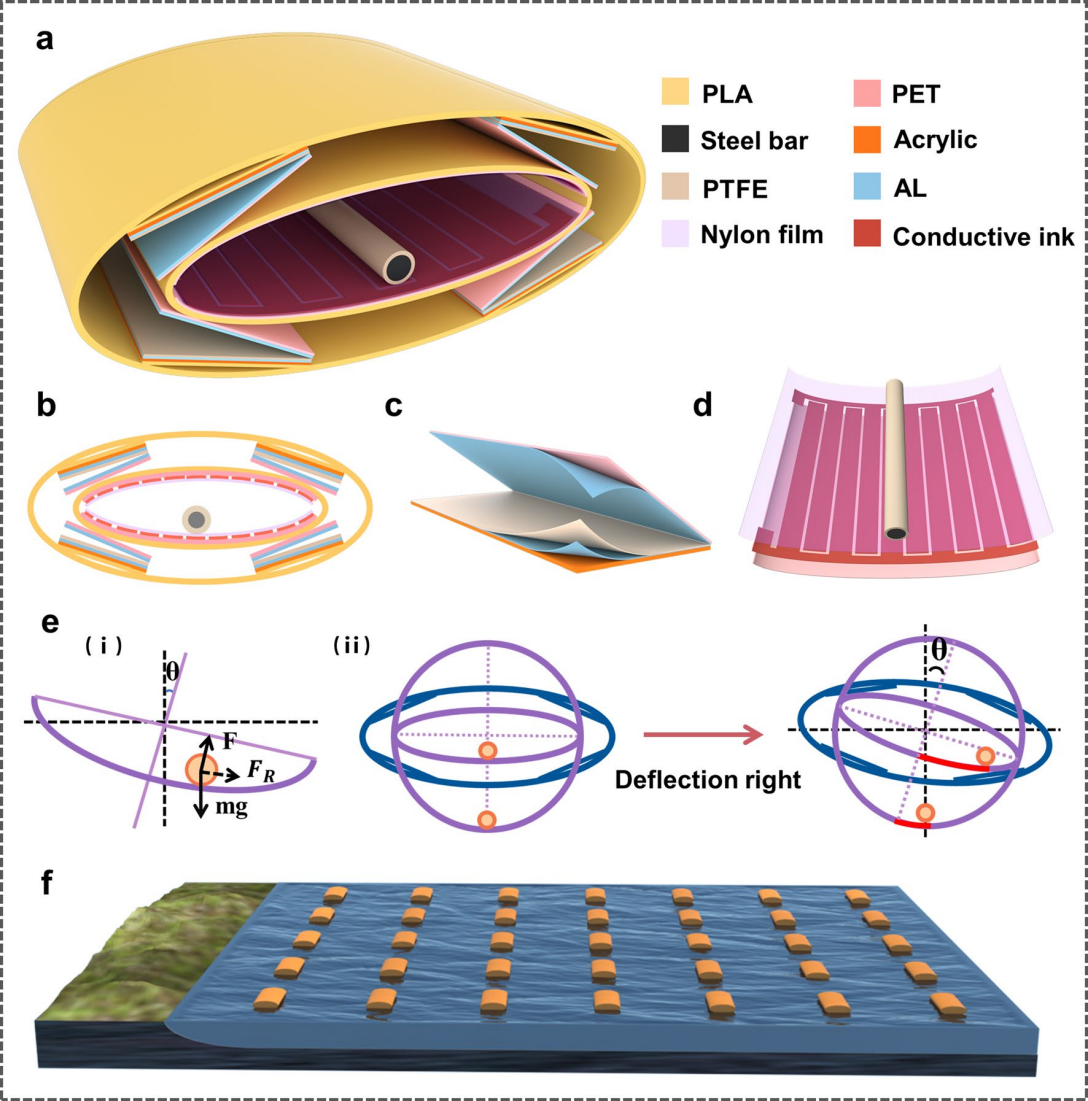
Structure design of the EC-TENG. **a** Schematic diagram of the EC-TENG. **b–d** Schematic diagrams of the cross-sectional configuration of EC-TENG, outer V-shaped TENG, and inner TENG. **e** Dynamic analysis of a steel bar rolling in the inner shell (i), and comparison of the elliptic cylindrical shell with cylindrical or spherical shells in bar rolling distance under the same slant angle (ii). **f** Proposed network composed of the EC-TENGs for large-scale blue energy harvesting

Figure 1a, b illustrates the detailed schematic design and cross-sectional view of the EC-TENG, respectively. As we can see, the EC-TENG adopts a completely symmetrical structure, employing two coaxial elliptical cylindrical shells as the structural support. Specifically, the internal cylindroid is a free-standing mode TENG, where two pairs of interdigital electrodes covered with Nylon films are symmetrically adhered to the lower and upper inner surfaces, and a steel bar wrapped with polytetrafluoroethylene (PTFE) is used as the rolling element. Four V-shaped TENGs with contact-separation mode are symmetrically fixed on the four corners of the gap between inner and outer elliptical cylindrical shells. The detailed structures of the outer V-shaped TENGs and internal TENG are schematically shown in Fig. 1c, d, respectively, and digital photographs of the as-fabricated device are shown in Fig. S1. Under wave excitation, the steel/PTFE bar can roll back and forth in the inner shell, which not only realizes the electrical output of the internal TENG but also provides the driving force for the contact-separation of the outer TENGs. The fabrication process for the EC-TENG is discussed in detail in the Experimental Section.

The device can operate in both tranquil and choppy seas is crucial important for a blue energy harvester. In previous works, various blue-energy harvesters have been demonstrated and TENGs with cylindrical and spherical structures have been studied the most [24, 44, 45]. However, when the sea is calm, it is difficult for most devices to respond to weak water waves. When the waves are rough, TENGs with cylindrical or spherical shell structures can easily flip over and lose their optimal working conditions. Therefore, in this work, we propose a TENG based on an elliptical cylinder structure. This novel design has two advantages. First, when the EC-TENG deviates from its equilibrium position under wave excitation and oscillates, the steel/ PTFE bar in the inner shell will be rolled by the combined action (F_R_) of gravity (mg) and support force (F), as illustrated in Fig. 1e (i). According to the comparison of the elliptical cylindrical shell in this work with the cylindrical or spherical shells with the same diameter as shown in Fig. 1e (ii), the bar goes farther in elliptical cylindrical shell under the same slant angle. Consequently, a larger effective working area can be achieved by the EC-TENG with improvement of its sensitivity to wave triggering and output performance. Thus, the tiny water wave energy available in tranquil seas can be effectively harvested. Meanwhile, the swing of the inner shell will drive the outer TENGs to contact and separate simultaneously. Second, an elliptical cylindrical structure is more difficult to be turned over compared to cylindrical or spherical structures, which enables the EC-TENG to possess unique self-stabilization and anti-overturning capability, ensuring a stable output at rough seas. Moreover, even if the entire device is flipped over under extreme conditions, its output will not be affected at all owing to its completely symmetrical structure, which distinguishes this work from other previous TENGs. Undoubtedly, the network of EC-TENGs can harvest large-scale ocean wave energy efficiently, so that it can serve as a robust power supply, as depicted in Fig. 1f.

**Fig. 2:**
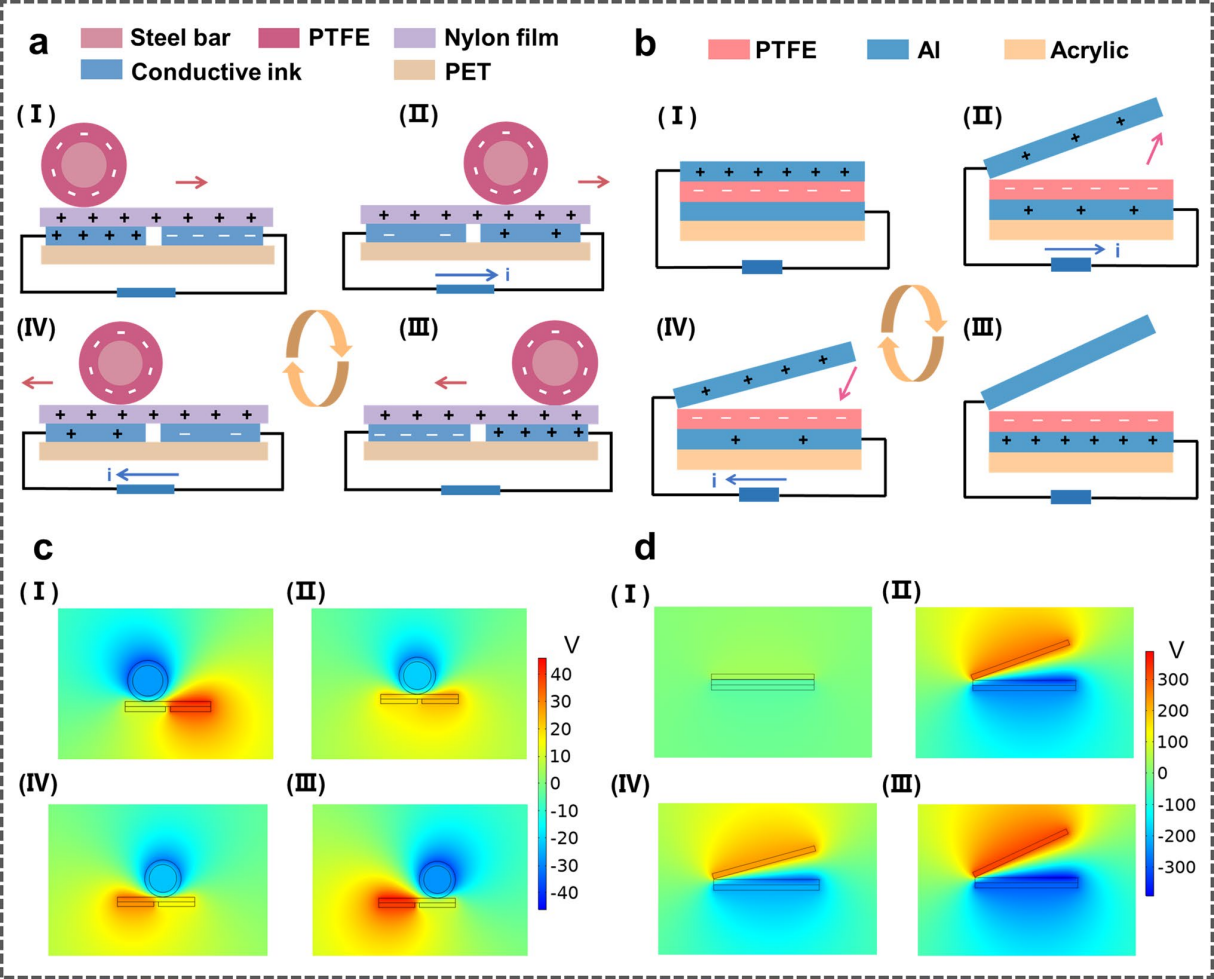
Working principles of the EC-TENG. **a, b** Sketches of the working principles of **a** the internal part and **b** the external part. **c, d** The corresponding potential distributions of the **c** inner TENG and **d** outer TENG calculated by COMSOL in a two-dimensional plane

The basic working principle of the EC-TENG is schematically plotted in Fig. 2, which can be elucidated from two aspects: the contact electrification and the electrostatic induction process [46, 47]. For the internal TENG (Fig. 2a), when the steel/ PTFE bar rolls under the excitation of an external wave, the surfaces of PTFE layer and Nylon film would be tribo-electrified, with static charges of the same amount and opposite signs. Accompanying the back and forth movement of the steel/PTFE rod, the negative charges on PTFE surface would induce electrons to flow between the two electrodes through an external circuit by utilizing the freestanding mode of TENG, generating alternate current in the circuit. The outer V-shaped TENG units demonstrate a similar energy generation process but utilize a contact-separation mode. As shown in Fig. 2b, when Al and PTFE come into contact with each other by the pressure of the inner shell, there will be equal amounts of triboelectric charges on the two surfaces based on the triboelectrification effect. An electric potential drop between the two electrodes will be built as long as the two tribo-layers are separated by the swing of the inner shell. To achieve static balance, the electrons will be driven to flow between the two electrodes because of the electrostatic induction. Furthermore, COMSOL Multiphysics software based on finite-element simulation is employed to calculate the potential distributions of the two TENGs at different states, as shown in Fig. 2c, d. The potential contour figures clearly depict the potential difference between the electrodes, which would drive the current flowing in the external circuit.

### Electrical Performance of the EC-TENG

**Fig. 3:**
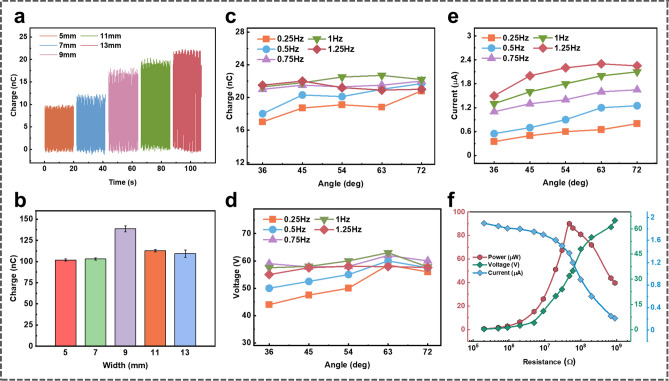
The electrical output performance of the internal TENG. **a** Transferred charge quantity under various electrode widths at *f* = 0.25 Hz and Deg = 27°. **b** The calculated transferred charge amount in a period under various electrode widths. **c–e** Point plots of the **c** transferred charge, **d** open-circuit voltage and **e** short-circuit current under diverse frequencies and swing amplitudes. **f** The output current, output voltage, and power dependence of the external load resistance at *f* = 1 Hz and Deg = 63°

To characterize the performance of the EC-TENG, we utilize a stepper motor to provide a reciprocating motion to simulate ocean wave movements and modulate the working frequency and pitch angle. First, the output performance of the inner TENG under different structure parameters and excitation conditions has been systematically investigated. The transferred charge quantity (*Q*_SC_) of the inner TENG with different interdigitated electrode widths is compared in Fig. 3a, and the corresponding open-circuit voltage (*V*_OC_) and short-circuit current (*I*_SC_) are shown in Fig. S2. Obviously, due to the increase of effective contact area, the output performance increases with the growth of electrode width. However, a larger electrode width will reduce the utilization rate of the device space, consequently reducing the number of output pulses per operating cycle. Therefore, the total transferred charge amount during one swing cycle with different electrode widths is calculated to determine the optimal electrode parameter. As shown in Fig. 3b, the *Q*_SC_ reaches the maximum at an electrode width of 9 mm, which equals the external diameter of the steel/PTFE rod. Thus, the electrode width is fixed at 9 mm for the following experiments.

To explore the capability of the inner TENG to capture wave energy under different ocean conditions, the influences of the oscillation frequency and pitch angle on the electric output are also explored. As illustrated in Fig. 3c–e, the inner TENG can operate at various swing frequencies (from 0.25 to 1.25 Hz) and pitch angles (36°–72°), demonstrating excellent feasibility of blue energy harvesting in both tranquil and choppy seas. Moreover, since the *Q*_SC_ and *V*_OC_ are mainly determined by the effective contact area of tribo-layers, they only increase slightly and fluctuate in a small range with the change of excitation conditions. In comparison, the *I*_SC_ is positively related to the operating frequency for ${I}_{\mathrm{SC}}=\frac{\mathrm{d}{Q}_{\mathrm{SC}}}{\mathrm{d}t}$. The optimal electric output is found at a pitch angle of 63° and a frequency of 1 Hz, showing a transferred charge of 22 nC, an open-circuit voltage of 63 V, and a short-circuit current of 2 μA. Under these optimal conditions, the output power of the inner TENG under various external resistances is further investigated, which reaches a maximum value of 90 µW with a matching resistance of 48 MΩ, as illustrated in Fig. 3f.

**Fig. 4:**
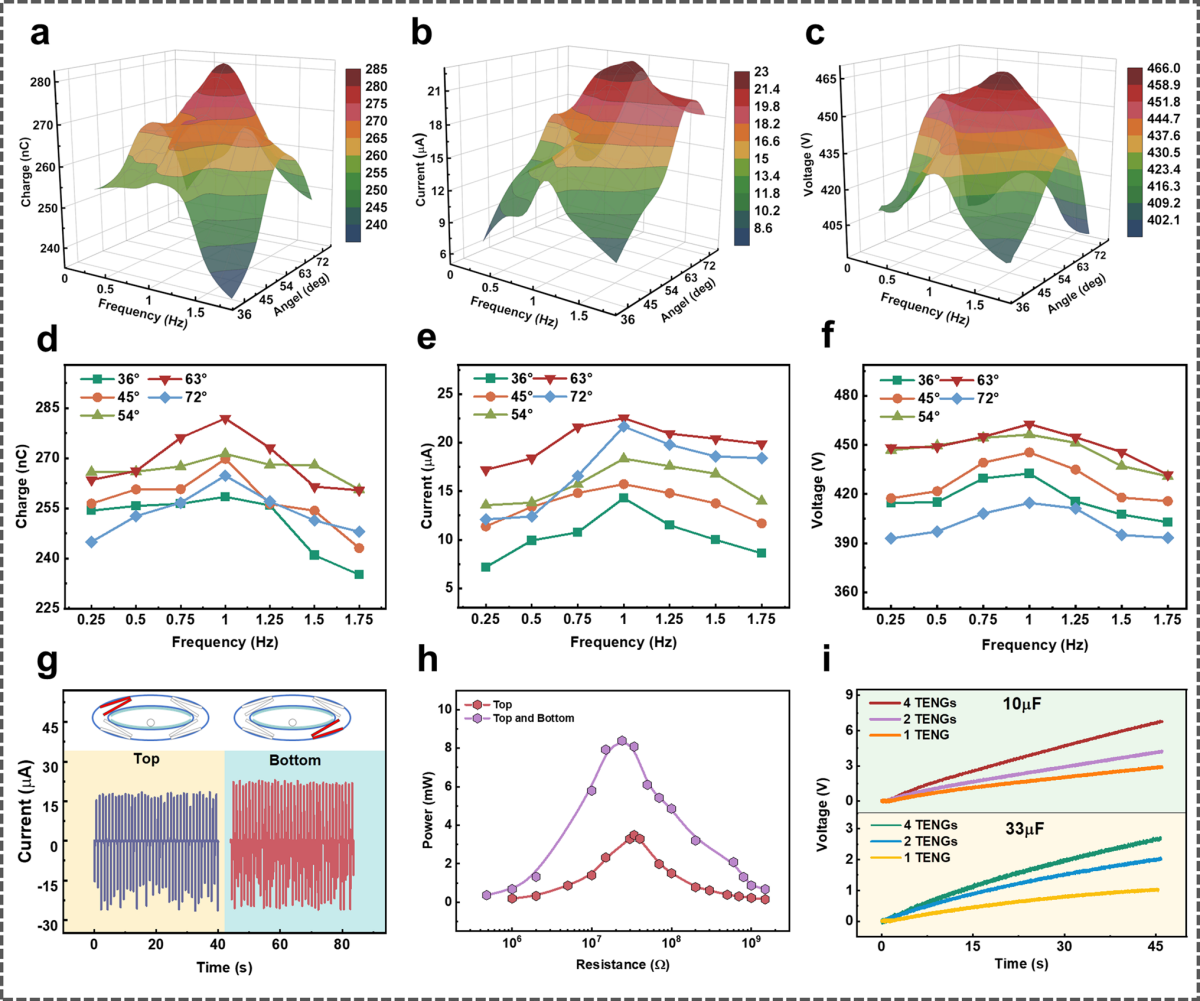
The electrical output performance of the outer TENG. **a–f** 3D surface graphs of **a** *Q*_SC_, **b** *I*_SC_, and **c** *V*_OC_ on changing both the working frequency and swing amplitude and their corresponding 2D graphs (**d–f**). **g** The short-circuit currents of a top and a bottom TENG at a frequency of 1 Hz and amplitude of 63°. **h** Peak power comparison of a single top TENG and a pair of diagonal TENGs connected in parallel (at 1 Hz and 63°). **i** Voltage curves of several commercial capacitors charged by different sets of outer TENGs (at 1 Hz and 63°)

The output behavior of the outer TENGs is another vital part of EC-TENG for blue energy harvesting. Since the outer TENGs are driven by the oscillation of the inner elliptical cylindrical shell, the units on the diagonals will work synchronously (achieve contact or separation at the same time). Herein, we take one of the bottom TENG units to further study the electrical performance, and the results are exhibited in Fig. 4a–f. As observed, the *Q*_SC_, *I*_SC_, and *V*_OC_ first increase and then decrease as the pitch angle and swing frequency increase. The reason that accounts for this phenomenon should be a larger vibration frequency or pitch angle can induce a more sufficient contact between the two tribo-layers. However, as the pitch angle or frequency increases further, the rapid swing of the inner shell causes the PTFE and Al films to separate quickly before achieving full contact, resulting in a drop in the output. The maximum amplitudes of the three signals are achieved at 1 Hz and 63°, where *Q*_SC_, *V*_OC_, and *I*_SC_ are approximately 280 nC, 460 V, and 22 μA, respectively.

In addition, the electrical performances of the TENGs along one diagonal are also investigated. As depicted in Figs. 4g and S3, under the optimal excitation condition (1 Hz and 63°), the two explored units (highlighted in red in the top inset of Fig. 4g) exhibit similar output features, but the bottom one delivers a higher output, which can be ascribed to the pressure from the inner TENG inducing a more adequate contact between the two tribo-layers. Moreover, since the motion states of the two diagonal TENGs are synchronized, they can be connected in parallel to achieve a higher output. As depicted in Figs. 4h and S4, under the same excitation condition (1 Hz and 63°), the peak power of a single top unit can only achieve 3.5 mW, while that of the diagonal TENGs connected in parallel can reach 8.3 mW. Additionally, three sets of TENG units, including a single bottom TENG, a pair of diagonal TENGs connected in parallel, and all four outer TENGs connected in parallel, are used to charge the commercial capacitors after rectifying. Obviously, better output performance and higher charging efficiency can be achieved by making full utilization of the four outer TENGs, as depicted in Fig. 4i.

The above results prove that the EC-TENG can convert the wave energy into electricity effectively in both tranquil (low frequency and pitch angle) and rough (high frequency and pitch angle) seas.

### Demonstration and Applications of EC-TENG

**Fig. 5:**
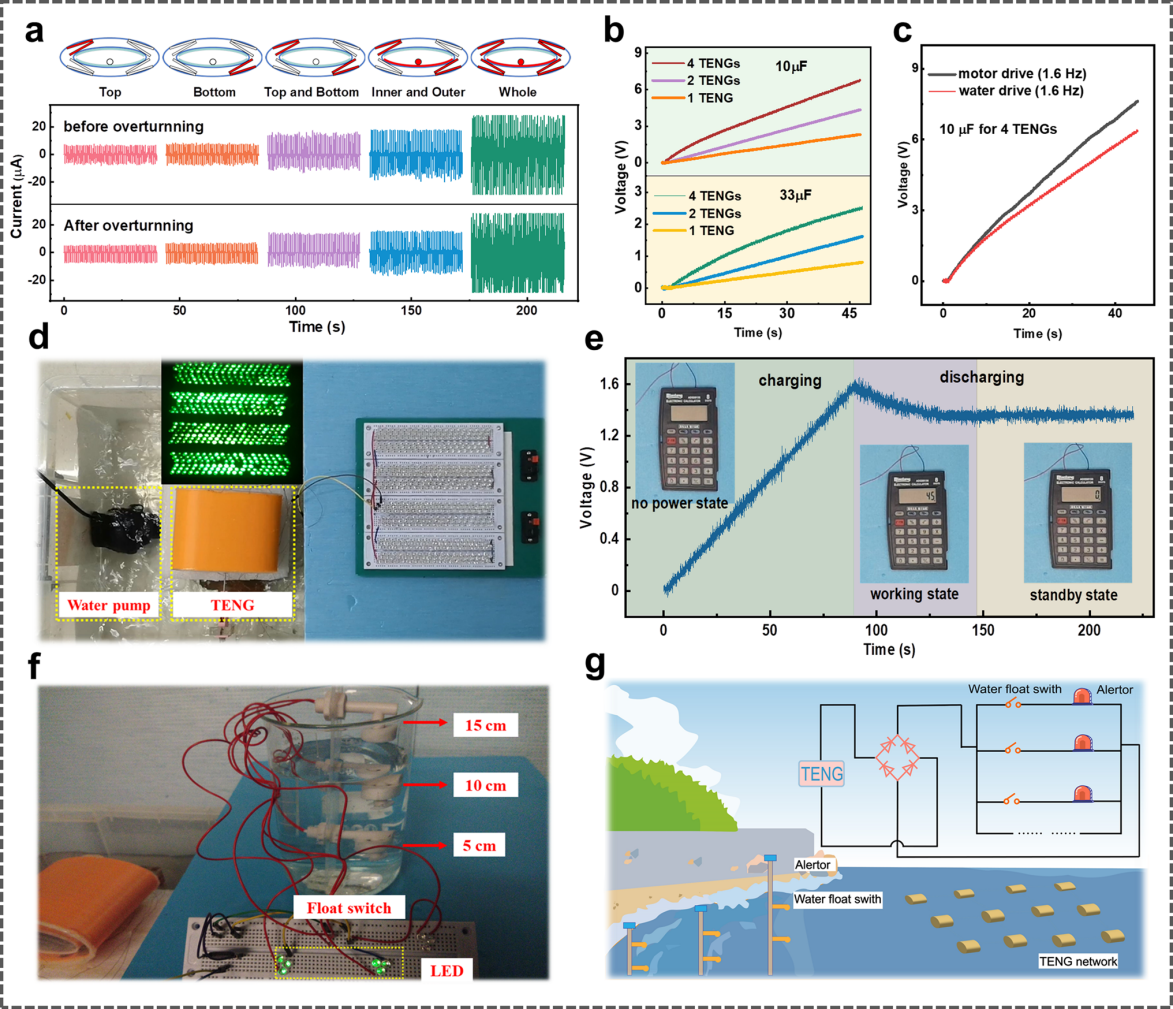
Demonstrations of the EC-TENG for wave energy harvesting. **a** Short-circuit current of the device before and after overturning. **b** Voltage profiles of several commercial capacitors charged by different sets of outer TENGs when the device is driven by a water wave. **c** Comparison of charging performances of stepper motor excitation and water wave triggering. **d** Image of 400 green LEDs lit by EC-TENG. **e** The voltage profile for an electronic calculator powered by the EC-TENG, where the insets show the working states of the electronic calculator. **f** Image of a water-level monitoring system powered by the EC-TENG. **g** A blueprint of the conceived EC-TENG-based water-level monitoring system for monitoring the coastal water levels, where the inset shows the circuit diagram of the water-level monitoring system

To explore the capability of the EC-TENG for real blue energy harvesting, a homemade water tank is employed to simulate the marine environment, and the EC-TENG is utilized to power various electronics under a simulated tide flow (frequency of 1.6 Hz). Figures 5a and S5 demonstrate the output values of different parts of the EC-TENG before and after overturning, while the top inset shows a cross-sectional view of the device, where the red parts are the corresponding units connected to the output. As can be seen, the device output does not change significantly after flipping over, indicating that the fully symmetrical structure ensures a steady performance of the EC-TENG under extreme ocean conditions, which is particularly important for practical applications of a blue energy harvester. Under a wave triggering, an optimal output power of 12 mW is delivered by the whole EC-TENG at a resistance of 30 MΩ, as illustrated in Fig. S6. The capacitor charging capability of the EC-TENG working in water is also investigated (Fig. 5b), and voltage values of the 10 and 33 μF capacitors can be raised to 6.3 and 2.5 V, respectively, within 45 s with the full utilization of 4 outer TENGs. Notably, the charging performance of the EC-TENG driven by a stepper motor is slightly higher than that triggered by a water wave due to the erratic motion of the real waves, as shown in Fig. 5c. Moreover, the EC-TENG is also demonstrated to light LEDs. As shown in Fig. 5d and Video S1, two diagonal outer TENGs and the internal TENG connected in parallel can successfully light up 400 green LEDs in series. With such an excellent performance in water, the EC-TENG has great potential to power various small electronic devices. In another practical application, a calculator driven by the EC-TENG is demonstrated by utilizing a 100 µF commercial capacitor to store the electric energy. As illustrated in Fig. 5e and Video S2, the capacitor can be charged to 1.6 V in 90 s and can then power a commercial calculator.

Furthermore, water level monitoring is of great importance in navigation, flood prevention and climate forecast. Herein, a self-powered water level monitoring and alarm system based on the EC-TENG is demonstrated. As depicted in Fig. 5f, the device has three water-level float switches aligned in different horizontal positions, including 5, 10, and 15 cm away from the bottom. Each float switch is connected in series with a set of corresponding LEDs and then connected in parallel to the rectified EC-TENG, as shown in the inset of Fig. 5g. When the water level rises to the relevant position, the switch will automatically close and the corresponding LEDs will be lit as an alarm signal. The detailed experimental process can be found in Video S3. For the potential large-scale application of this system, the EC-TENG network can be installed near the shore to harvest water-wave energy, while the switches can be arranged at different locations along the shore to monitor the water level in real-time, as shown in Fig. 5g. These results imply that the EC-TENG can be used not only for wave energy harvesting but also for building self-powered systems.

## Conclusions

In summary, a fully symmetrical EC-TENG based on an elliptic cylindrical structure is proposed for all-weather blue-energy harvesting. This innovative design demonstrates several superior attributes. First, benefiting from the elliptical cylindrical configuration, the EC-TENG exhibits high sensitivity in small agitations, excellent self-stability, and anti-overturning characteristics in rough seas. Second, the fully symmetrical design enables the device to produce a stable output even when flipped over under extreme conditions. Third, the inner and outer TENGs can work synchronously, which not only fully utilizes the device space but also helps to achieve a higher output in practical applications. The output behavior of the EC-TENG under different input conditions has been studied comprehensively, which indicates the device can harvest wave energy efficiently in both rough and tranquil seas. Under a real water wave triggering, the EC-TENG is capable of directly driving 400 LEDs, charging commercial capacitors, and powering an electronic calculator. Moreover, a water-level monitoring system based on the EC-TENG has been successfully demonstrated, which can effectively monitor the water level and provide an alarm in real-time. This work provides a novel structure that might inspire new designs for TENGs for large-scale blue energy harvesting.

## Supplementary Information

Below is the link to the electronic supplementary material.

Supplementary file1 (PDF 580 kb)

Supplementary file2 (MP4 8513 kb)

Supplementary file3 (MP4 15681 kb)

Supplementary file4 (MP4 8730 kb)
