# Supplementary material for: Anti-Overturning Fully Symmetrical Triboelectric Nanogenerator Based on an Elliptic Cylindrical Structure for All-Weather Blue Energy Harvesting
Source: Nanomicro Lett. 2022 May 11;14:124. doi: 10.1007/s40820-022-00866-w (PMC9095809; doi:10.1007/s40820-022-00866-w)
Supplement: Supplementary file 1 — Supplementary file1 (PDF 580 kb) [file 40820_2022_866_MOESM1_ESM.pdf]

Supporting Information for

Anti-Overturning Fully Symmetrical Triboelectric Nanogenerator Based on an Elliptic Cylindrical Structure for All-Weather Blue Energy Harvesting

Dujuan Tan[1], Qixuan Zeng[1], Xue Wang[1, *], Songlei Yuan[1], Yanlin Luo[1], Xiaofang Zhang[1], Li ming Tan[1], Chenguo Hu[1], and Guanlin Liu[1, 2, *]

[1] Department of Applied Physics, State Key Laboratory of Power Transmission Equipment & System Security and New Technology, Chongqing University, Chongqing, 400044, PR China
[2] Center on Nanoenergy Research, School of Physical Science and Technology, Guangxi University, Nanning, Guangxi 530004, P. R. China

*Corresponding authors. E-mail: xuewang@cqu.edu.cn (X. Wang), guanlinliu@gxu.edu.cn (G.L. Liu)

Supplementary Figures

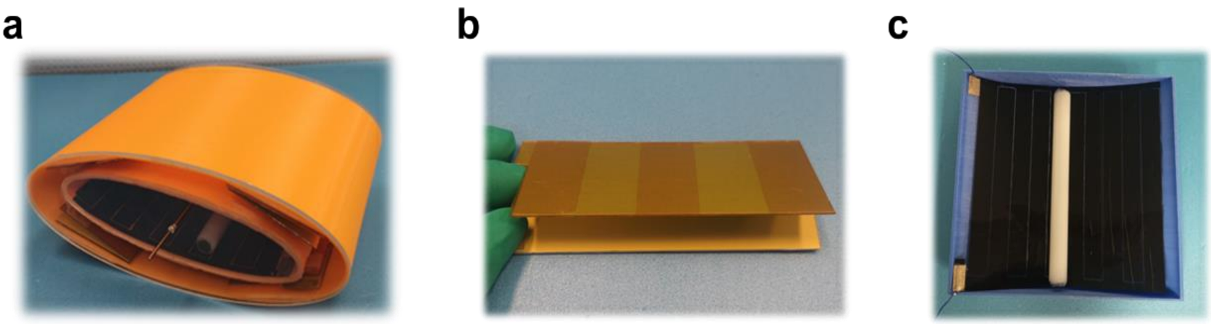

Fig. S1 Photographs of (**a**) as-fabricated EC-TENG device, (**b**) a V-shaped outer TENG, and (**c**) the inner TENG

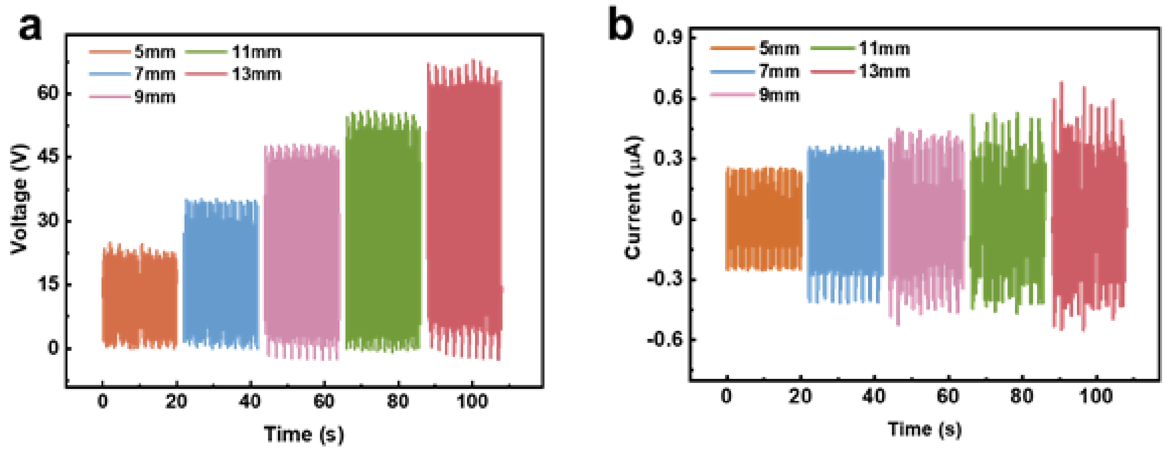

Fig. S2 (**a**) Open-circuit voltage and (**b**) short-circuit current of the inner TENG under various electrode widths at f = 0.25 Hz and Deg = 27°

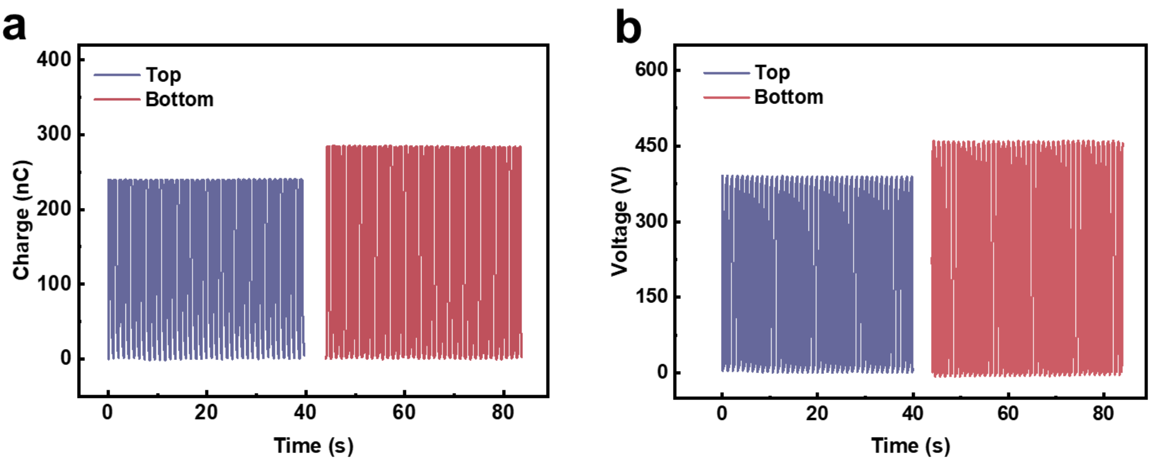

Fig. S3 (**a**) Transferred charge and (**b**) open-circuit voltage of a top and a bottom outer TENG driven by stepper motor under f = 1 Hz and Deg = 63°

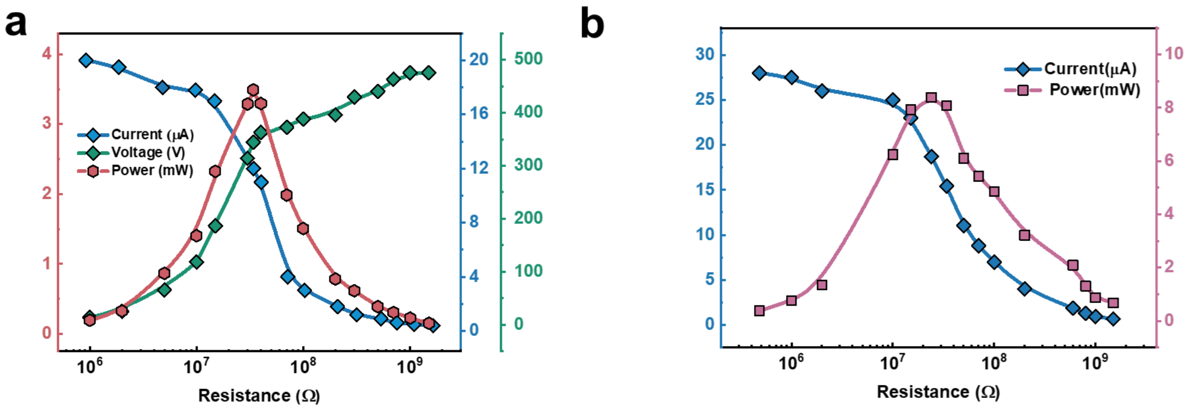

Fig. S4 Output power of (**a**) a single top TENG and (**b**) two diagonal TENGs in parallel under different external load resistances (f = 1 Hz and Deg = 63°)

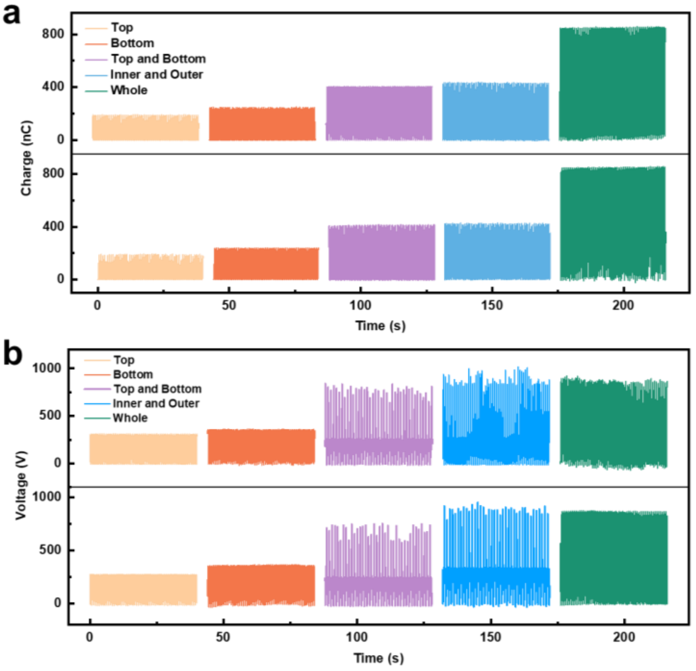

Fig. S5 Output performance of EC-TENG excited by water waves (1.6 Hz): (**a**) transferred charge and (**b**) open-circuit voltage of EC-TENG before and after overturning

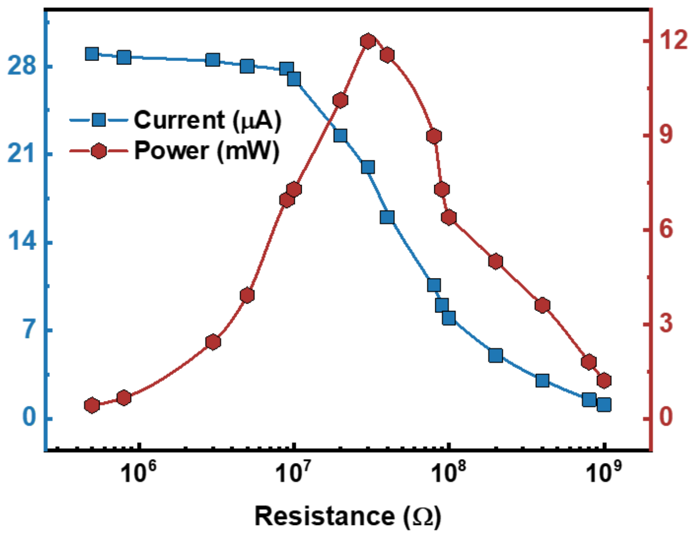

Fig. S6 Output power of whole EC-TENG under different external load resistances when the device is driven by a water wave (f = 1.6 Hz)

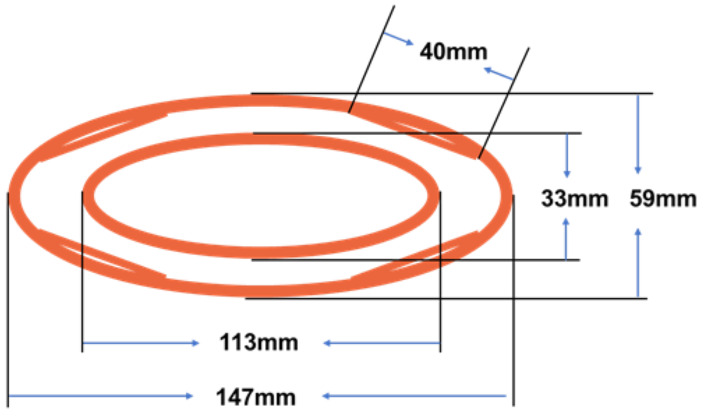

Fig. S7 Parameters of inner and outer elliptic cylindrical shells

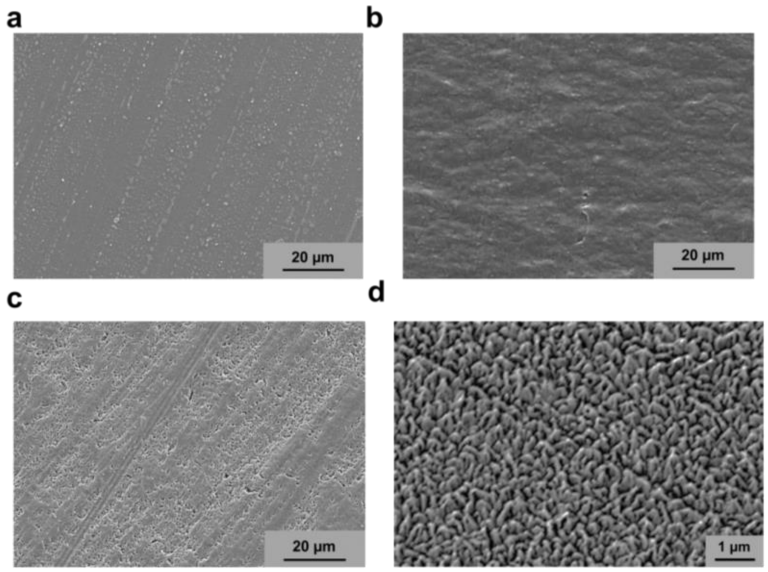

Fig. S8 SEM images of triboelectric material surfaces: (**a**) nylon film, (**b**) aluminum film, (**c**) pristine PTFE film, (**d**) PTFE film after surface treatment
